# Intracranial Metastases Originating From Pediatric Primary Spinal Cord Glioblastoma Multiforme: A Case Report and Literature Review

**DOI:** 10.3389/fonc.2020.00099

**Published:** 2020-02-11

**Authors:** Dengpan Song, Dingkang Xu, Qiang Gao, Peizhu Hu, Fuyou Guo

**Affiliations:** ^1^Department of Neurosurgery, The First Affiliated Hospital of Zhengzhou University, Zhengzhou, China; ^2^Department of Pathology, The First Affiliated Hospital of Zhengzhou University, Zhengzhou, China

**Keywords:** spinal cord glioblastoma, intracranial metastases, pediatric, case report, literature review

## Abstract

Primary spinal cord glioblastoma multiforme (scGBM) is an uncommon entity in pediatrics, and intracranial metastasis originating in spinal cord gliomas is very rare. A 7-year-old female presented with weakness in the limbs, paralysis of the lower limbs and incontinence. The initial MRI of the spinal cord revealed expansion and abnormal signals from T2 to T5. She was initially diagnosed with Neuromyelitis optica spectrum disorders and treated with high-dose glucocorticoid and gamma globulin. Four months later, her symptoms worsened and follow-up imaging showed multiple intracranial mass lesions. We performed a subtotal resection of the right thalamic basal ganglia tumor and gross total resection of the right frontal lobe tumor under microscopic examination. Histopathology revealed scGBM with intracranial metastasis and the molecular pathology diagnosis suggested H3K27M mutant diffuse midline glioma WHO grade IV, which had previously been misdiagnosed as a Neuromyelitis optica spectrum disorders. We review the literature of intracranial metastases originating from pediatric primary spinal cord glioblastoma multiforme and summarize possible methods of differentiation, including changes in muscle strength or tone, intramedullary heterogeneously enhancing solitary mass lesions and cord expansion in MRI. Finally, we emphasize that in unexpected radiological changes or disadvantageous response to the treatment, a biopsy to achieve a pathological diagnosis is necessary to discard other diseases, especially neoplasms.

## Introduction

Glioblastoma multiforme (GBM) is the most common primary CNS tumor in adults but it is not common in children. However, among the group of malignant glial tumors, high grade glioma is the most frequent diagnosis in children. Furthermore, the incidence of intramedullary tumors is very rare in children; most are low grade gliomas (1). A spinal cord origin of glioblastoma in both adults and children is rare (2). Metastatic intracranial GBM disseminating from a primary intramedullary spinal GBM is extremely uncommon and has rarely been reported in the world literature. Here, we review the literature (3–20) on intracranial metastases originating from primary scGBM in pediatric patients (Table 1). Surgical resection followed by radiotherapy and concomitant adjuvant chemotherapy is the recommended treatment in children.

**Table 1:** Review of the literature on intracranial metastases originating from primary spinal cord glioblastoma multiforme in pediatric patients.

**Case no**.	**References**	**Age(years)/Sex**	**Level of tumor**	**Surgery**	**RT**	**CT**	**Survival (mos)**
1	O'connell et al. (17)	16/M	Conus	Biopsy	Y	N	16
2	Tashiro et al. (16)	12/F	Conus	STR	N	N	11
3	Johnson et al. (15)	8/F	T11-L3	STR	Y	Y	14
4	Cohen et al. (20)	17/F	Thoracic	NR	Y	NR	10
5		16/F	Conus	NR	Y	NR	6
6		14/M	Conus	NR	Y	NR	4
7		10/F	Cervical	NR	Y	NR	5
8		9/M	Cervical	NR	Y	NR	1
9	Allen et al. (14)	4/M	Holocord	STR	N	Y	37
10		5/M	Thoracic	Biopsy	Y	Y	68
11		15/M	Cervical	STR	Y	Y	48
12	Klepstad et al. (19)	12/F	Cervical	STR	NR	NR	2
13	Kawashima et al. (13)	8/F	C7-T11	Surgery	Y	Y	12
14	Caroli et al. (12)	6/M	T9-T11	STR	Y	Y	9
15	Stecco et al. (11)	14/M	Conus	STR	N	N	10
16	Battaglia et al. (9)	11/M	T4-T5	GTR	Y	Y	6
17	Bonde et al. (10)	16/M	Conus	GTR	Y	N	6
18	Sun et al. (8)	14/M	Conus	STR	N	N	16
19	Kim et al. (18)	16/F	T12-L1	Biopsy	Y	Y	12
20	Mori et al. (7)	10/F	Conus	Biopsy	Y	Y	14
21	Derinkuyu et al. (6)	9/F	T8-T10	Surgery	Y	Y	8
22	Kokkalis et al. (5)	12/M	T4-T8	GTR	Y	Y	20
23	Kumar et al. (3)	4/M	Cervicothoracic	Biopsy	Y	Y	4
24	Yan et al. (4)	10/M	T11-L1	GTR	Y	Y	14
25	The present case	7/F	T2-T5	STR	N	N	1

*NR, not reported. The data were obtained from PubMed*.

This report presents an uncommon case of scGBM with intracranial metastasis closely mimicking Neuromyelitis optica spectrum disorders in both symptoms and MRI findings. The unique features of MRI and the clinical symptoms are summarized in the present article.

## Case Report

**Figure 1:**
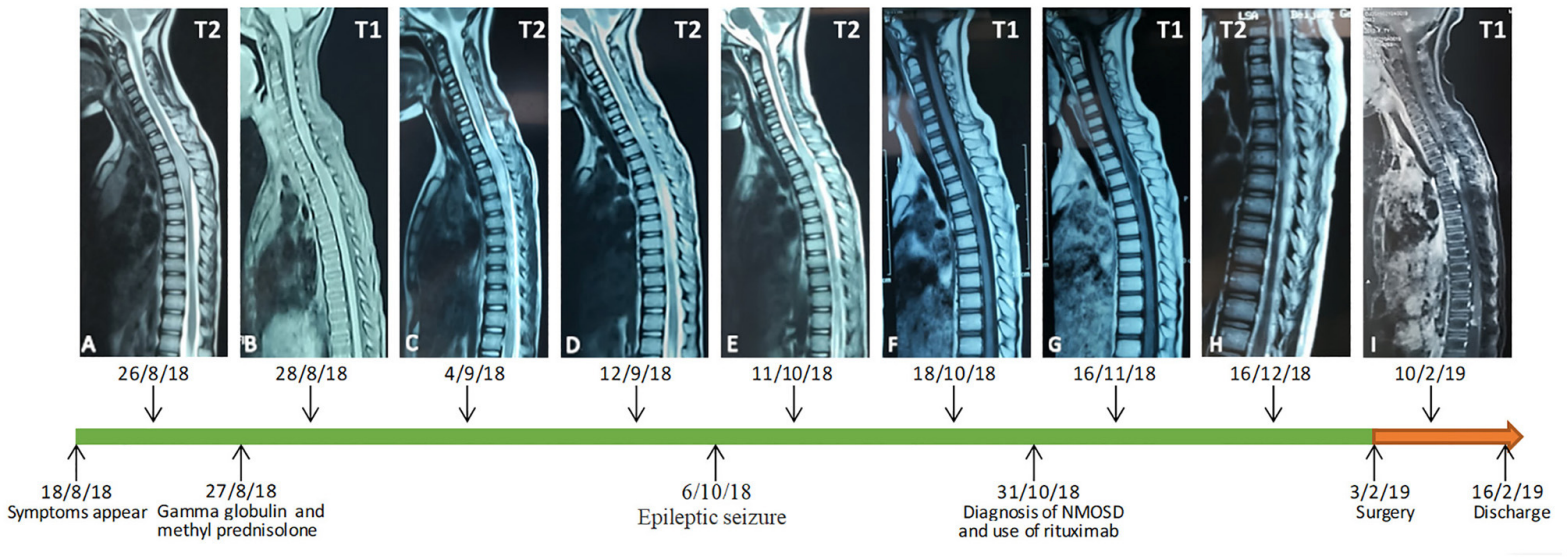
An early MRI examination showed expansion and abnormal signals from T2 to T5 **(A,B)**. Seven days later, a spinal MRI showed the lesions had become enlarged **(C,D)**. A follow-up MRI showed multiple abnormal signals in the cervicothoracic spinal cord, and the T1 to T6 level spinal cord was enlarged and showed a long T2 signal **(E,F)**. The abnormal signal range and the thickening of T1 to T6 level crista medulla had slightly decreased **(G)**. One month later, the abnormal area of the signal was enlarged, and the signal was enhanced **(H)**. In a postoperative spinal contrast enhanced MRI, there were multiple signals showing heterogeneous enhancement in the medulla oblongata, the c3-7 level cervical spinal cord and its margin, the t3-6 level spinal canal, and the t2-l1 level spinal cord and its margin. Patchy enhancement was observed in the soft tissue behind the thoracolumbar vertebrae, the bilateral cerebellum, the anterior pontine cistern and the fourth ventricle **(I)**.

**Figure 2:**
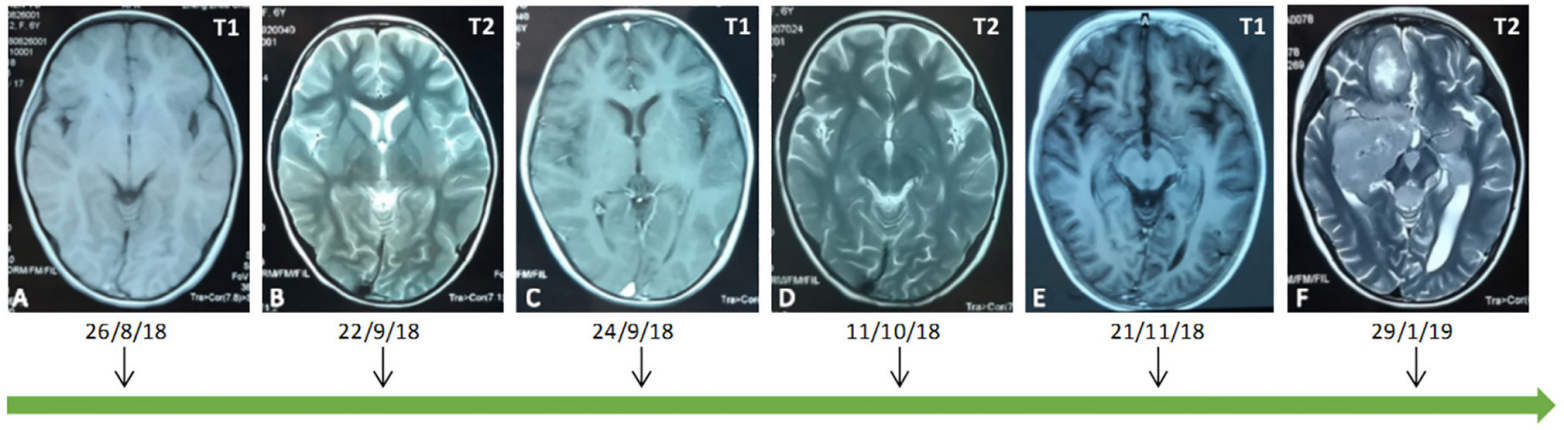
An early MRI showed that the brain parenchyma was normal **(A**–**C)**. Over 2 weeks later, an MRI showed that the right occipital lobe, bilateral parietal lobe, right thalamus, right hippocampus and temporal lobe showed a sheet equal signal on T1 and a long signal on T2 **(D,E)**. Preoperatively, on a brain MRI examination, abnormal signals were found in bilateral cerebellum, anterior cistern of pons, right cerebellopontine angle, fourth ventricle, bilateral hippocampus, right basal ganglia, bilateral paraventricles, right frontal lobe, left temporal parietal lobe, pineal region, left thalamus, and left cerebral peduncle **(F)**.

A 7-year-old female patient was admitted to the hospital because of a 7-days history of weakness in her left lower limb, intermittent chest pain, and aggravation at night. The patient presented with a fever of more than 39 degrees centigrade, urinary and fecal incontinence and bilateral limb paralysis after 16 days, and her temperature was normal after high-dose glucocorticoid and gamma globulin intensive therapy, due to the suspicion of acute transverse myelitis. Gamma globulin (2 g/kg) and methyl prednisolone (20 mg/kg) were administered for 4 days. She underwent lumbar puncture, and a cerebrospinal fluid (CSF) assay showed that the protein level was 4073.4 mg/l. Then, her symptoms deteriorated in her upper limbs, and the patient had a seizure. Imaging examinations and urine culture analysis confirmed a diagnosis of pneumonia and urinary tract infection. She received meropenems and cephalosporins, but the neurological symptoms did not improve. Then, considering the mild remission from the initial intensive therapy, gamma globulin (2 g/kg) and methyl prednisolone (20 mg/kg) were administered again 5 days later. The CSF examination showed a growing CD19-positive T and B cell count (815 U/L). She was diagnosed with a Neuromyelitis optica spectrum disorder and was treated with gamma globulin (2 g/kg) on day 10 and Rituximab 0.3 mg on day 15. Her spinal cord and brain MRI findings revealed changes in accordance with the course and severity of the disease (Figures 1, 2).

The patient presented to our institution with a 4-months history of weakness in her limbs. Her physical examinations showed bilateral lower limb muscular tension with an Ashworth Grade of 1; right and left upper limb muscle strength with grades of 4/5 and 3/5, respectively; bilateral lower limb muscle strength with a grade of 0/5; hypesthesia of superficial sense below C2 (mainly in the left limbs); non-cooperation on a deep sense examination; hyperfunctional bilateral biceps and triceps tendon reflexes (++); a hyperfunctional bilateral patellar and Achilles tendon reflexes (+++) with a grade of 3/4; a bilateral ankle clonus (+); and a positive bilateral Babinski's sign (+), which was suggestive of brain involvement. A brain contrast-enhanced MRI examination showed multiple intracranial lesions (Figure 2).

**Figure 3:**
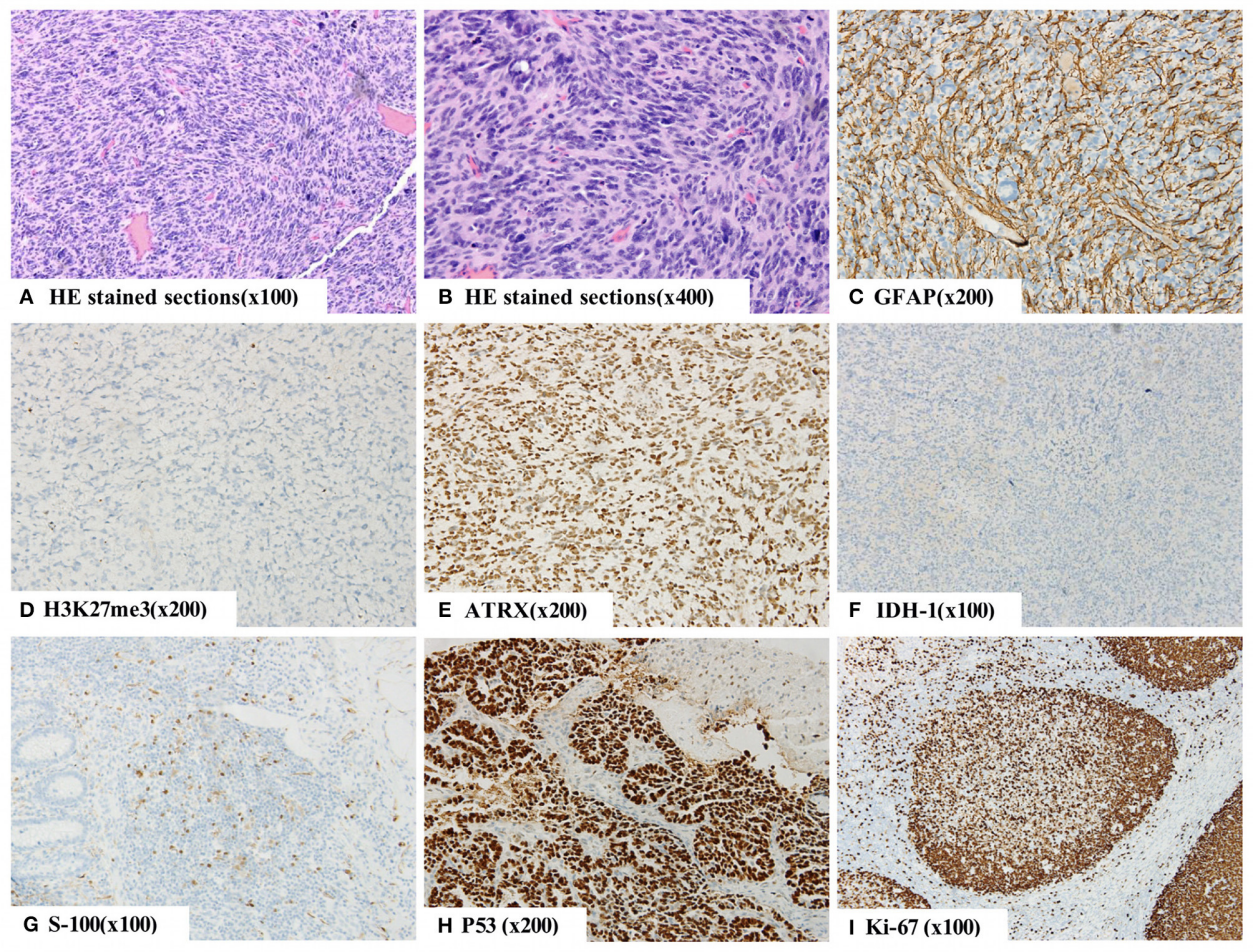
The histopathology of right basal nodule and frontal lobe indicating a malignant tumor **(A,B)**. By immunohistochemistry, the tumor cells were positive for GFAP **(C)**, S-100 protein **(G)**, Oligo-2, ATRX **(E)**, P53 **(H)**, SOX-10, Syn, CD56, and INI-1 and negative for IDH-1 **(F)**, NeuN and H3K27me3 **(D)**. Moreover, they were weakly positive for CD99, and 60% of them were positive for Ki-67 **(I)**. The final diagnosis was WHO grade IV glioblastoma (GBM), and the molecular pathology diagnosis was H3K27M mutant diffuse midline glioma WHO grade IV.

We suspected a high-grade glioma and performed subtotal resection of the right thalamic basal ganglia tumor and gross total resection of the right frontal lobe tumor under microscopic examination. The pathological diagnosis was WHO grade IV GBM, and the molecular pathology diagnosis was H3K27M mutant diffuse midline glioma WHO grade IV (Figure 3).

Postoperatively, the patient was treated with antiepileptic drugs and neurotrophic drugs, such as injection of mouse nerve growth factor. On a postoperative spinal MRI, there were multiple signals showing heterogeneous enhancement in the medulla oblongata, at the c3-7 level of the cervical spinal cord and margin, at the t3-6 level of the spinal canal, and at the t2-l1 level of the spinal cord and its margin. Patchy enhancement was observed in the soft tissue behind the thoracolumbar vertebrae, the bilateral cerebellum, the anterior pontine cistern and the fourth ventricle.

Eleven days after the operation, the patient developed status epilepticus and coma. Despite conservative treatment, her general condition and neurological status did not improve. Her relatives asked that she be discharged 2 days later, and she died a month later.

## Discussion

High-grade gliomas (HGGs) are the most common group of pediatric malignant CNS neoplasms, and midline tumor location in children is more frequent than in adults (21). As far as primary spinal glioblastoma, Beyer et al. found that it mainly affect younger patients, and the median age at diagnosis in their cohort was 22 years (2). However, the incidence of primary spinal cord glioblastoma multiforme (scGBM) in the pediatric age group is still very rare (22). The most common pattern of progressive disease is local recurrence, and intracranial metastasis of spinal cord gliomas is extremely rare (21). Here, we review the literature (3–20) on intracranial metastases originating from primary scGBM in pediatric patients (Table 1). Fifty-six percent of the children were male and 44% were female. The median OS was 10 months. Thirty-two percent of the tumors were conus, 28% were thoracic, 16% were cervical, 12% were thoracolumbar, 8% were cervicothracic, and 4% were at a holocord location. Regarding surgery type, subtotal resection was performed in 36% of the cases, and biopsy was achieved in 20%. GTR was performed in 16% of the cases and 8% mentioned only surgery. Regarding treatment type, 20% of the children underwent surgery only. Forty-eight percent received both RT and CT after surgery, 28% received only RT after surgery, and 4% received only CT after surgery. Several factors are associated with the tendency of a tumor to disseminate; these include a young age, a high histology grade, cellular anaplasia, oligodendroglial differentiation, overexpression of epidermal growth factor receptor, increasing Ki-67 values and immunosuppressed states (23–25), some of which were found in our case. Intracranial seeding of spinal cord glioma may occur due to dissemination of the tumor into the spinal subarachnoid space and subsequent intracranial spread into the cerebral subarachnoid space or brain parenchyma via the CSF (26, 27). Hence, we believe that it is necessary to perform cytologic examinations of the CSF so that the metastasis can be discovered earlier, even when no associated abnormal CSF is found, as occurred in our case.

MRI is an essential auxiliary diagnostic method that helps when judging the location of a spinal tumor and is very useful and helpful for tumor differentiation. The patient's tumors always showed mixed hypo-isointense signals on T1-weighted images (T1WI) and hyperisointense signals on T2-weighted images (T2WI). On MRI with contrast, scGBM presents with an irregular zone of contrast enhancement, and could be expressed as intramedullary heterogeneously enhancing solitary mass lesions and cord expansion in children (28). With the development of the disease, this performance will become more obvious, and there may be local or diffuse metastasis. The majority of intramedullary tumors in children are astrocytomas and ependymomas (29), with ependymomas characterized by a high incidence of syringomyelia and homogeneously enhanced solid tumor sections. Low-grade astrocytoma usually lacks significant reinforcement after Gd-DTPA is applied. However, because of the imaging non-specificity and rarity of scGBM, it is difficult to diagnose this type of lesion based on MRI alone. It can mimic an epidural pathology, in some cases including an epidural abscess (30), Neuromyelitis optica spectrum disorders or lymphoma on contrast MRI. Therefore, in the case of some special spinal cord diseases that are difficult to recognize based on clinical and imaging findings, a biopsy and pathological examination should be performed to avoid misdiagnosis.

Spinal GBM is identified based on pathology parameters, such as its intracranial counterparts, including nuclear atypia, mitotic activity, vascular proliferation, and necrosis (28, 30), as well as immunohistochemical parameters, such as GFAP and S-100 positivity with a high Ki-67 leveling index (28, 31), the latter of which was observed in the intracranial lesions in our case. While the histology of HGGs between adults and children appears identical, the biology of the tumors may vary significantly. The mutation rate of P53 in children with GBM was 40%. Overexpression of p53 was significantly associated with dramatically reduced progression-free survival (PFS) at 5 years, while mutations in TP53 were non-significantly associated with adverse prognosis (32). Mutations of IDH-l and IDH-2 have a low incidence in primary GBM, and high-grade glioma (HGG) with IDH gene mutation has a significantly better prognosis (33). The promoter methylation rate of MGMT was 40–57% in GBM, and the methylation of the MGMT promoter suggests that the prognosis of patients with GBM is better in both adults and children, while GBM with MGMT promoter methylation may be sensitive to alkylating agent (32, 34). Only 5.0% of cases of glioblastoma multiforme were associated with 1p/19q codeletion, and Clark et al. (35) confirmed that 1p/19q testing is not useful on gliomas that are histologically GBM. A total of 15.3% of enrolled GBM cases demonstrated loss of ATRX expression (ATRX-), and the adult patients with ATRX- showed better survival than patients with counterpart proteins expressed in GBM (36). In addition, H3K27me3 was negative in molecular pathology, suggesting that H3K27M mutation may be present; thus, the final molecular pathology diagnosis may be H3K27M mutant diffuse midline glioma WHO grade IV. The diagnosis of WHO grade IV GBM is mainly based on routine histopathology. Overall survival was significantly shorter when the H3K27M mutation was present in HGG, and HGG patients positive for the H3K27M mutation are more than three times more susceptible to succumbing to this disease by more than 2 years, compared to patients negative for the mutation (37). Therefore, the presence of H3K27M mutation may suggest H3K27M mutant diffuse midline glioma and indicate a high metastasis rate and poor prognosis.

Surgery is still the primary treatment for spinal GBM and related intracranial metastasis. However, there is controversy regarding the surgical extent of resection that should be achieved in high-grade spinal glioma patients. Ononiwu et al. (38) noted that children treated with GTR survived longer on average than those treated with STR. However, Konar et al. (22) found that extent of resection does not have a direct relationship with OS. Moreover, the surgical procedure should also be selected in consideration of the preservation of neural function. In some cases of late-stage scGBM, the tumor is too close to the functional nerve area to be separated completely, and in these cases, partial resection should be performed while preserving the original nerve function. We believe that total resection should not be performed solely to prevent recurrence but that quality of life should also be improved and survival time maximized, both of which can be achieved by precise subtotal resection with preservation of neural function. In the case presented here, to remove as many tumors as possible without compromising safety and improve the quality of life of patient, we performed a total microscopic resection of the right frontal lobe tumor and extended the resection to the glial hyperplasia zone. Nevertheless, the right thalamic basal ganglia tumor was resected only subtotally to avoid serious dysfunction due to its ill-defined boundary and infiltration of conduction tracts.

The role of radiotherapy and chemotherapy in GBM is still much debated. In their study of 158 high-grade spinal cord gliomas, Jiang Lui et al. (39) found that the use of postoperative RT offered a survival benefit, especially in pediatrics (22). Minehan et al. (40) found that postoperative RT substantially enhanced overall survival in high grade gliomas. However, Lam et al. (41) found that increased RT for spinal GBM does not prolong OS in pediatrics. Similarly, reports about the effects of chemotherapy have produced conflicting results. Some reviews have not demonstrated a significant difference in long-term survival between groups treated with or without temozolomide (TMZ) (42). Nevertheless, some studies have found that the TMZ improved prognoses in both adults and children (22, 43). With regard for radiotherapy, we do not suggest that it may cause a deterioration in medullary function, edema and reduced quality of life. In addition, gene-based detection and molecular targeted therapy may also play a role in GBM (44).

Neuromyelitis optica spectrum disorders (NMOSDs) are rare autoimmune diseases in which NMO-IgG selectively binds to aquaporin-4 (AQP4). NMOSDs mainly involves the optic nerve, spinal cord and central nervous system in the form of an inflammatory response in these areas. According to the new NMOSD diagnostic criteria, we suggest some characteristics that may be used to differentiate them: (1) serum AQP4 antibody detection; (2) association with clinical symptoms and MRI findings typical of optic neuritis; (3) in NMOSD, few muscles are involved, while scGBM often manifests with changes in muscle strength or tone, such as progressive weakness or paresthesia in both lower limbs; (4) a fast-moving process is frequently observed in NMOSD; and (5) rapid and significant improvement after high-dose glucocorticoid treatment. However, due to similarities in clinical symptoms and imaging findings and the rarity of both diseases, we were unable to accurately differentiate scGBM and NMOSD. There were some major potential contributors to the misdiagnosis that occurred in our case: (1) the nonspecific presentation of symptoms and imaging findings, (2) the rapid progression of the disease over time, and (3) the slight improvement observed after glucocorticoid treatment. This improvement may have been due to the remission of the secondary inflammation of the tumor rather than the treatment for NMOSD. There are very few records of scGBM imitating NMOSD. We emphasize the necessity of biopsy or surgical pathology in differentiating between spinal cord inflammation and neoplastic disease.

While acute transverse myelitis (ATM) is a relatively common disease, it is rare in children. Compared with scGBM, ATM may present with substantially higher protein levels in CSF (mean value of 17,375 g/dL and range 45–1,120 g/dL) and a notable absence of T1 hypointensity on spinal MRI (45), but these are not diagnostic. Because the imaging findings of ATM are always relatively variable and non-specific, MRI plays only a differential role in some typical cases. In the present case, a rise in the CSF protein level and slight improvement in the patient's condition were observed, and these may have been influenced by the secondary inflammation of the tumor, affecting the doctor's diagnosis and causing the misdiagnosis. Therefore, it is crucial to perform surgical biopsy or surgical pathology in indistinguishable cases.

Primary central nervous system lymphoma (PCNSL) is a rare aggressive non-Hodgkin lymphoma confined to the brain, spine, eyes, and leptomeninges. It represents only 4% of intracranial neoplasms, and 4–6% of extranodal lymphomas. Phenotypically, PCNSL is almost always a diffuse large B-cell lymphoma (DLBCL) (46). This disease is very rare in pediatric patients, and its clinical features and treatment outcomes in this group are not well-understood. (47). Primary CNS lymphoma is almost non-existent in pediatric-aged patients and should not be taken into account in the differential diagnosis in such cases.

## Conclusion

Intracranial metastases originating from pediatric primary scGBM are extremely rare, and their atypical symptoms and imaging findings are the main causes of their misdiagnosis as other conditions, such as NMOSD, ATM, or lymphoma. Therefore, we should fully consider the possibility of scGBM and pay attention to differential diagnoses. If a patient's clinical symptoms and MRI and other auxiliary examinations do not allow a definitive diagnosis, it is necessary to perform a biopsy or surgical pathology as soon as possible.

## Data Availability Statement

All datasets generated for this study are included in the article/supplementary material.

## Ethics Statement

The protocol was approved by the medical ethical committee of Zhengzhou University. This patient's parents gave written informed consent. The authors affirm that written informed consent was obtained from the participant for the publication of this case report.

## Author Contributions

DS: manuscript writing. DX: conception and critical review. QG and PH: image extraction and pathological review. FG: conception and critical review. All authors proofread and approved the manuscript.

### Conflict of Interest

The authors declare that the research was conducted in the absence of any commercial or financial relationships that could be construed as a potential conflict of interest.
